# Subchronic exposure to titanium dioxide nanoparticles modifies cardiac structure and performance in spontaneously hypertensive rats

**DOI:** 10.1186/s12989-019-0311-7

**Published:** 2019-06-24

**Authors:** Stefano Rossi, Monia Savi, Marta Mazzola, Silvana Pinelli, Rossella Alinovi, Laura Gennaccaro, Alessandra Pagliaro, Viviana Meraviglia, Maricla Galetti, Omar Lozano-Garcia, Alessandra Rossini, Caterina Frati, Angela Falco, Federico Quaini, Leonardo Bocchi, Donatella Stilli, Stéphane Lucas, Matteo Goldoni, Emilio Macchi, Antonio Mutti, Michele Miragoli

**Affiliations:** 1 Department of Medicine and Surgery, University of Parma, Via Gramsci, n° 14, 43126 Parma, Italy; 2 CERT, Center of Excellence for Toxicological Research, INAIL, ex-ISPESL, University of Parma, Parma, Italy; 3 Department of Chemistry, Life Sciences and Environmental Sustainability, University of Parma, Parma, Italy; 4 Institute for Biomedicine, Eurac Research, Bolzano, Italy; 5 Affiliated Institute of the University of Lübeck, Lübeck, Germany; 6 Namur Nanosafety Centre (NNC), Namur Research Institute for Life Sciences (NARILIS), Research Centre for the Physics of Matter and Radiation (PMR), University of Namur, B-5000 Namur, Belgium; 7 Azienda Ospedaliera-Universitaria, Unità di Medicina del lavoro e Tossicologia industriale, Parma, Italy; 8 Humanitas Clinical and Research Center, Rozzano, Milan, Italy; 9 Present address: Cátedra de Cardiología y Medicina Vascular, Escuela de Medicina y Ciencias de la Salud Tecnologico de Monterrey, Monterrey, Mexico; 10 Present address: Department of Biomedical and Neuromotor Sciences, University of Bologna, 40126 Bologna, Italy

**Keywords:** Titanium dioxide nanoparticles, Nanotoxicology, Cardiac electrophysiology, Cardiac fibrosis, Arrhythmias

## Abstract

**Background:**

Non-communicable diseases, intended as the results of a combination of inherited, environmental and biological factors, kill 40 million people each year, equivalent to roughly 70% of all premature deaths globally. The possibility that manufactured nanoparticles (NPs) may affect cardiac performance, has led to recognize NPs-exposure not only as a major Public Health concern, but also as an occupational hazard. In volunteers, NPs-exposure is problematic to quantify. We recently found that inhaled titanium dioxide NPs, one of the most produced engineered nanomaterials, acutely increased cardiac excitability and promoted arrhythmogenesis in normotensive rats by a direct interaction with cardiac cells. We hypothesized that such scenario can be exacerbated by latent cardiovascular disorders such as hypertension.

**Results:**

We monitored cardiac electromechanical performance in spontaneously hypertensive rats (SHRs) exposed to titanium dioxide NPs for 6 weeks using a combination of cardiac functional measurements associated with toxicological, immunological, physical and genetic assays.

Longitudinal radio-telemetry ECG recordings and multiple-lead epicardial potential mapping revealed that atrial activation times significantly increased as well as proneness to arrhythmia. At the third week of nanoparticles administration, the lung and cardiac tissue encountered a maladaptive irreversible structural remodelling starting with increased pro-inflammatory cytokines levels and lipid peroxidation, resulting in upregulation of the main pro-fibrotic cardiac genes. At the end of the exposure, the majority of spontaneous arrhythmic events terminated, while cardiac hemodynamic deteriorated and a significant accumulation of fibrotic tissue occurred as compared to control untreated SHRs. Titanium dioxide nanoparticles were quantified in the heart tissue although without definite accumulation as revealed by particle-induced X-ray emission and ultrastructural analysis.

**Conclusions:**

The co-morbidity of hypertension and inhaled nanoparticles induces irreversible hemodynamic impairment associated with cardiac structural damage potentially leading to heart failure. The time-dependence of exposure indicates a non-return point that needs to be taken into account in hypertensive subjects daily exposed to nanoparticles.

**Electronic supplementary material:**

The online version of this article (10.1186/s12989-019-0311-7) contains supplementary material, which is available to authorized users.

## Background

Communicable and non-communicable diseases increase at the same rate in low and high-income countries occurring among the same patient populations, and it is fundamental to define an effective and sustainable action [1]. For example, the World Heart Federation counts 970 million people suffering from high blood pressure that is one of the most important causes of death with a still growing trend. Many questions involving the effects of engineered nanomaterials on the environment, as well as human health, have been raised and need to be addressed [2]. Manufactured nanoparticles have become a new component of the air we breathe [3]. Since the heart and the lungs are intimately linked, it is difficult to identify the specific susceptibility of either organ to the effects of nanoparticles (NPs) [4]. It is possible to better define the impact of particle matter (PM) on individual organ impairment or disease [5, 6] through the use of animal models. Recently, in order to further clarify the relationships between fine particles and cardiopulmonary diseases, the spontaneously hypertensive rat (SHR) has been widely used as a model of cardiovascular hypertension for researches on toxicology and pathogenesis of cardiovascular adverse effects of exposure to PM [7]. Limited experimental evidence from cardiopulmonary-compromised animal models has shown increased mortality from chronic exposure to simulated particle/gaseous urban air pollution in SHR [8]. Chronic systemic hypertension and associated cardiomyopathy may increase the susceptibility to PM-induced morbidity. In fact, in control SHR the ECG showed a depression in ST-segment area which was not present in Wistar Kyoto (WKY) rats and a further depression in ST-segment area in SHR during the first and second days of PM exposure. This enhanced depression was less evident following 3 days of exposure, perhaps suggesting adaptive changes [9].

The first study to report long-term cardiovascular toxicity of an inhaled engineered nanomaterial, clearly demonstrated that long-term exposure to inhaled nickel NPs can induce oxidative stress and inflammation, not only in the lung but also in the cardiovascular system [10]. Recent data showed that long-term silica inhalation in rat, at realistic concentrations, induced very low systemic toxicity and negligible pulmonary fibrogenicity [11].

Titanium dioxide (TiO_2_) is one of the most widely produced engineered nanomaterials. As consumption grows the chance of population exposure to fine or ultrafine TiO_2_ increases. In 2007 the National Institute for Occupational Safety and Health (NIOSH) estimated 68.000 workers in U.S. directly in contact with TiO_2_ pigments, with an estimated production of TiO_2_ of 1.45 million metric tons / year (https://www.cdc.gov/niosh/docs/2011-160/pdfs/2011-160.pdf) and the demand of TiO_2_ increases exponentially worldwide. Compared to the high risk of heart disease by PM exposure, the TiO_2_ effects are still partially known [12]. It is estimated 1.56 billion hypertensive people by 2025 [13]; nowadays, one third of the population between 15 and 69 years old suffer from high blood pressure, while half of them is either not conscious of the hypertension nor undergoes medical treatment [14]. It is thus considerable that a similar percentage of workers daily exposed to TiO_2_-NPs suffers of hypertension. In 2011, the NIOSH conducted a systematic review of TiO_2_ health effects from epidemiologic, animal, and in vitro studies and defined a quantitative risk assessment. In fact, exposure limits of 2.4 mg/m^3^ for fine TiO_2_ and 0.3 mg/m^3^ for ultrafine TiO_2_ were recommended, as time-weighted average concentrations for up to 10 h per day during a 40-h work/week [15]. Subjects with underlying health issues such as asthma and hypertension may be at increased risk of TiO_2_-NPs toxicity [16]. A significant association between inhalable TiO_2_-NPs exposure and declines in pulmonary function has been demonstrated, as well as increase in blood pressure, especially in small airways functions and systolic blood pressure in healthy workers at the end of shift [17]. Exposure of rats, by intra-tracheal instillation, to a well-dispersed suspension of ultra-fine TiO_2_-NPs caused dose-dependent pulmonary damage and inflammation, which persisted 42 days post-exposure [2]. Another work demonstrated that exposures to nano sized TiO_2_ had no significant long-term adverse pulmonary effects, even if acute levels of inflammation and cytotoxicity were observed for TiO_2_-NPs, at the highest dose of 5 mg/kg, 1 day post-exposure [18]. We recently showed that a single acute intra-tracheal instillation of TiO_2_-NPs in normotensive animals, induces cardiac electrophysiological alterations especially in the repolarization phase, in both isolated cardiomyocytes and in vivo rat heart, giving rise to a pro-arrhythmic substrate [19] due to the direct formation of nano pores across cell membrane [20]. It also has been demonstrated that, when SHR and WKY rats were exposed to the same dose of fine particles including metallic elements such as Fe, Mg, Zn, Pb and Cu, the lung injury in SHR was greater than in WKY rats, due to an increase in cytokines [21], and rats with cardiopulmonary diseases were more susceptible than healthy rats [22].

The aim of the present study was to investigate the effects of subchronical exposure to TiO_2_-NPs on cardiac structure and electromechanical performance in SHRs, being aware that TiO_2_-NPs can rapidly translocate from the lung into the bloodstream and finally to the heart [19]. Here, we demonstrated a pathological cardiac remodeling associated with an increment of spontaneous arrhythmic events mainly including sinus pauses and atria-ventricular blocks (AV blocks), changes in conduction velocity, and worsened hemodynamic performance. Interestingly, our approach identified a time-point characterized by cardiac and pulmonary inflammatory response, nanotoxicity and up-regulation of genes deputed to control collagen deposition in the heart, ultimately resulting in an irreversible increment of diffuse cardiac fibrosis compared to the untreated SHRs.

## Results

**Fig. 1:**
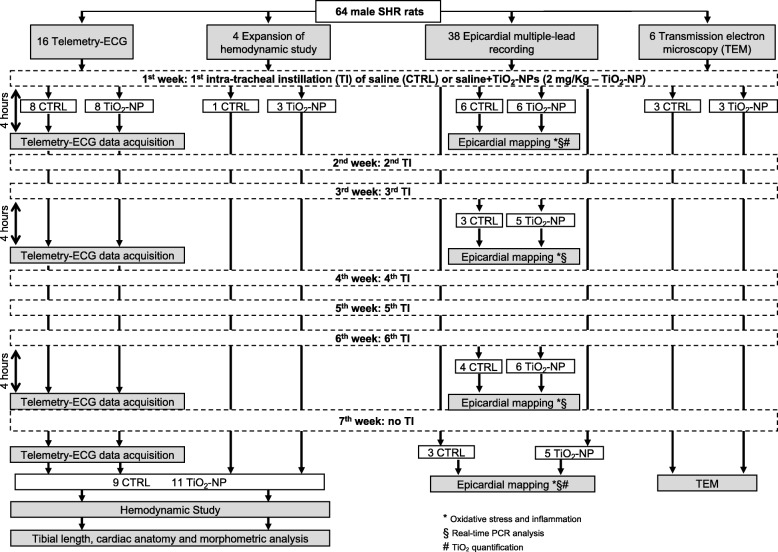
Study population and experimental protocol. Flowchart describing the number of animals subjected to the different experimental procedures before and after intra-tracheal instillation of saline solution (CTRL group) or saline solution added with TiO_2_, at a final concentration of 2 mg/kg (TiO_2_-NP group). * timepoints for oxidative stress and inflammation; § timepoints for real-time PCR analysis; # timepoints for PIXE. For more details see “Outline of the experimental protocols” section

The experimental design and measurements performed in the various animal groups are summarized in Fig. 1.

### Telemetry-ECG study

**Fig. 2:**
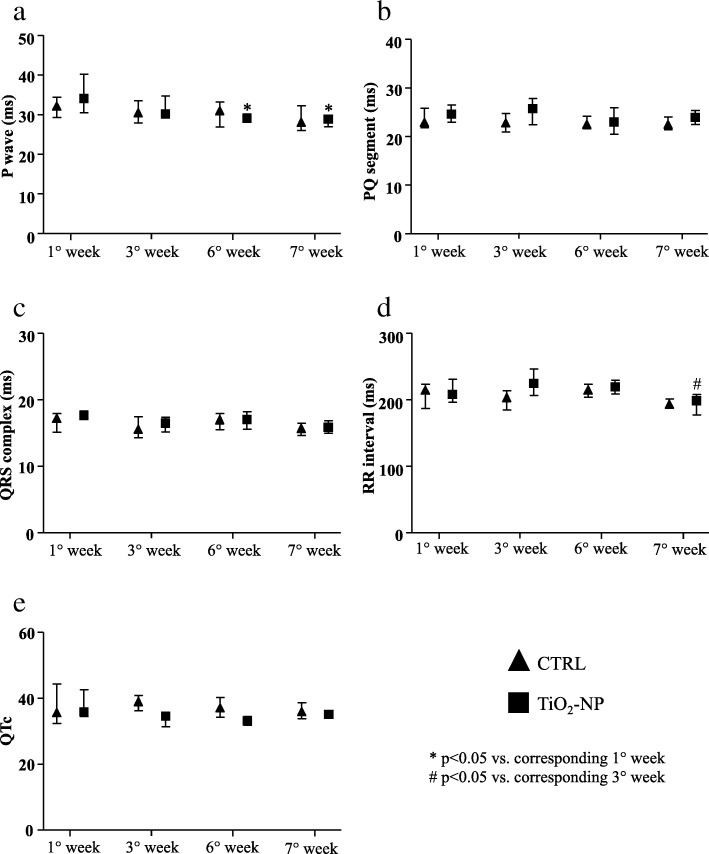
Electrocardiographic waveform and interval durations. Basic electrophysiological parameters evaluated in CTRL (triangles) and TiO_2_-NPs treated (square) animals. **a** P wave duration (ms). **b** PQ segment duration (ms). **c** QRS complex duration (ms). **d** RR interval duration (ms). **e** QTc duration (ms). Kruskal-Wallis (post hoc analyses: Dunn’s multiple comparison) was performed and statistical significance was set at *p* < 0.05. * vs corresponding 1° week; # vs corresponding 3° week. Data are represented as median and interquartile range (IQR)

Electrocardiographic wave and interval durations showed only time-dependent effects as illustrated in Fig. 2. Atrial activation (P wave duration) in NPs exposed SHRs was reduced at the 6th week and the end of the longitudinal protocol (7th week) compared to the first week (Fig. 2a). We did not observe significant differences for PQ segment, QRS complex and QTc (Fig. 2b, c and e). RR interval was significantly reduced only at the end of the experimental protocol in the treated animals (Fig. 2d).

**Fig. 3:**
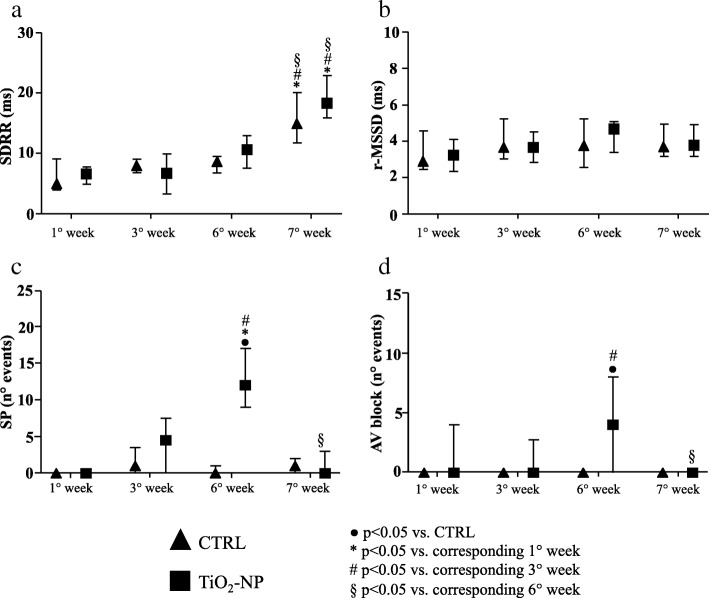
Heart rate variability indexes and arrhythmia evaluation. Heart rate variability parameters and spontaneous arrhythmic events in CTRL (triangles) and TiO_2_-NP (square) groups. **a** SDRR duration (ms). **b** r-MSSD duration (ms). **c** Sinus pauses (SP, number of events). **d** Atrio-ventricular blocks (AV block, number of events). Kruskal-Wallis (post hoc analyses: Dunn’s multiple comparison’s) was performed and statistical significance was set at *p* < 0.05. ● vs CTRL; * vs corresponding 1° week; # vs corresponding 3° week; § vs corresponding 6° week. Data are represented as median and IQR

The heart rate variability, evaluated as standard deviation of RR interval (SDRR), increased only at final stage in both groups (Fig. 3a), while no differences were seen in the root mean square of successive RR interval square differences (r-MSSD) value (Fig. 3b). ECG-telemetry analysis revealed different types of arrhythmic events (Additional file 1: Figure S1). Arrhythmia quantification demonstrated that sinus pauses and AV blocks significantly increased in TiO_2_-NPs group in comparison with control (CTRL) group only after the 6th instillation (nine and sixteen-fold increase, respectively; Fig. 3c and d). This increase disappeared when TiO_2_ treatment was suspended. Instead, supraventricular and ventricular arrhythmias were similar in the two experimental groups during the entire protocol (data not shown).

### Hemodynamic study

The heart rate measured under anesthesia was similar in both groups as well as systolic and diastolic arterial blood pressure, and left ventricular systolic pressure (LVSP) whose values were coherent with the degree of hypertension expected in 19-week old SHR (Table 1). Compared with CTRL animals, TiO_2_-NP rats exhibited a global deterioration of hemodynamic performance, as indicated by the significant increase in the left ventricular (LV) end-diastolic pressure (+ 28%, LVEDP, Table 1), the decrement in the maximal rate of LV pressure rise (− 15%, +dP/dt_max_, Table 1) and decline (− 15%, −dP/dt_max_, Table 1), and the marked prolongation of isovolumic contraction time (+ 10%, IVCT, Table 1).

**Table 1:** Hemodynamic study

	CTRL (*n* = 9)	TiO_2_-NP (*n* = 11)
Heart rate (beats/min)	188 ± 5	190 ± 5
Systolic arterial BP (mmHg)	197.5 ± 2.12	198.16 ± 7.08
Diastolic arterial BP (mmHg)	122.4 ± 4.36	123.0 ± 4.92
LVSP (mmHg)	195.9 ± 2.90	195.4 ± 5.96
LVEDP (mmHg)	6.51 ± 0.55	8.32 ± 0.64*
+dP/dt_max_ (mmHg/s)	9639.6 ± 86.37	8190.1 ± 264.5*
-dP/dt_max_ (mmHg/s)	− 7790.6 ± 117.7	− 6608.2 ± 91.28*
IVCT (s)	0.020 ± 0.0002	0.022 ± 0.0003*

Values are means ± standard error of the mean (SEM)

Hemodynamic measurements were performed at 7th week, before sacrifice. Unpaired Student’s t-test

*BP* blood pressure, *LVSP* left ventricular systolic pressure, *LVEDP* left ventricular end-diastolic pressure, *+dP/dt*_*max*_ maximal rate of left ventricular (LV) pressure rise, *−dP/dt*_*max*_ maximal rate of LV pressure decline, *IVCT* isovolumic contraction time

*p < 0.05 vs CTRL

### Epicardial mapping study

**Fig. 4:**
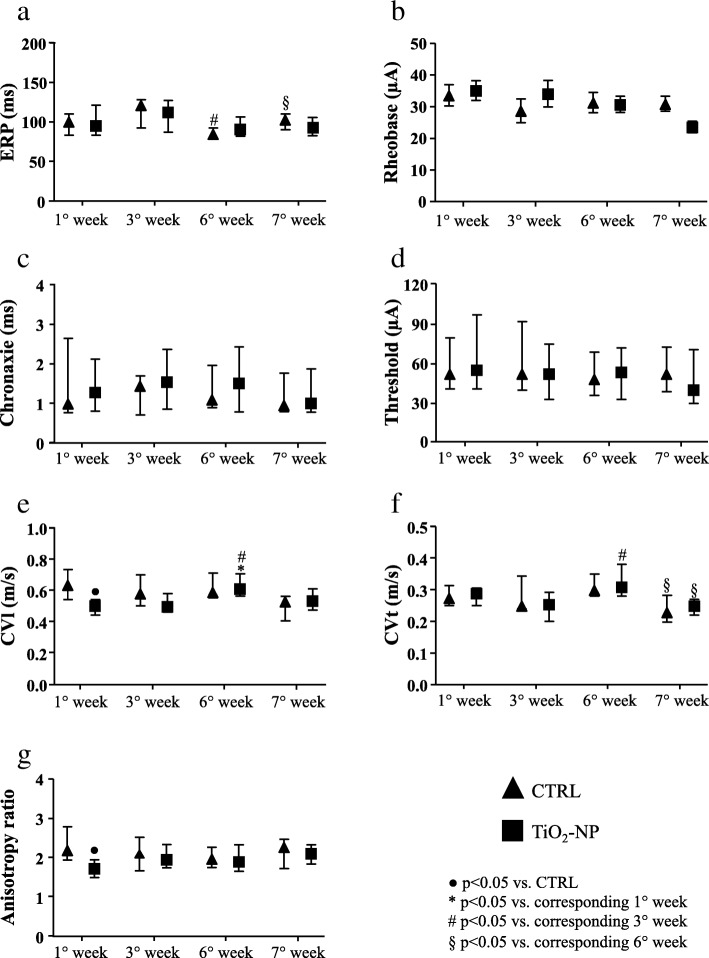
Cardiac refractoriness, excitability, anisotropic conduction velocities and arrhythmia induction. Electrophysiological parameters and the index of arrhythmia inducibility in CTRL (triangles) and TiO_2_-NPs treated (squares) animals. **a** Effective refractory period (ERP, ms). **b** Rheobase (μA). **c** Chronaxie (ms). **d** Threshold intensity for a 1 ms duration impulse (μA). **e** ventricular conduction velocities along fiber (CVl, m/s). **f** ventricular conduction velocities across fiber (CVt, m/s). **g** ventricular conduction velocities ratio (CVl/CVt). Kruskal-Wallis (post hoc analyses: Dunn’s multiple comparison) was performed and statistical significance was set at *p* < 0.05. ● vs CTRL; * vs corresponding 1° week; # vs corresponding 3° week; § vs corresponding 6° week. Data are represented as median and interquartile range (IQR)

In-vivo electrophysiological parameters were evaluated by means of epicardial multiple leads recording. Cardiac excitability remained unchanged (Fig. 4b-d) during the entire protocol while refractoriness decreased at the 6th week in the CTRL group and then completely recovered (7th week) to the values measured at the beginning of the experimental protocols (Fig. 4a). As compared to CTRL, the treatment induced a significant decrease of ventricular conduction velocity as well as anisotropy ratio by about 25% after first instillation (Fig. 4e and g). Afterwards the differences between the two groups disappeared. A time-dependent increment of longitudinal conduction velocity (CVl) was observed in the TiO_2_-NP group at the 6th week. After 1 week of recovery (7th week) CVl and transverse conduction velocity (CVt) decreased in both groups (Fig. 4e and f). Arrhythmia inducibility in TiO_2_-NP rats displayed an increasing significant trend till 6th week reducing its value at the end of experimental protocol after 1 week of suspended treatment (data not shown).

### Tibial length, cardiac anatomy and morphometric analysis

**Fig. 5:**
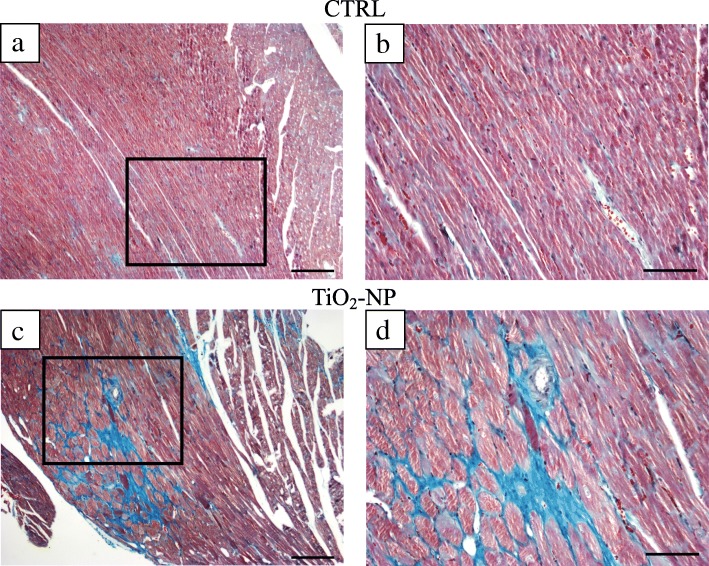
Cardiac tissue fibrosis evaluation. Masson’s trichrome staining sections were analyzed by optical microscopy to evaluate perivascular and interstitial fibrosis in the LV myocardium (greenish). Representative images of heart sections from control (**a** and **b**) and TiO_2_-NP treated animals (**c** and **d**). **a** CTRL LV myocardium; black rectangle area is shown at higher magnification in panel (**b**). **c** TiO_2_-NPs treated LV myocardium; black rectangle area is shown at higher magnification in panel (**d**). Scale bars: **a** and **c** = 200 μm; **b** and **d** = 100 μm

Body weight (BW) at sacrifice was similar between the two groups, while tibial length and left ventricular weight (LVW) were significantly reduced in TiO_2_-NP rats (− 7% for both parameters; Table 2) showing a reduced growth. LV wall thickness, chamber diameter, chamber length, and LVW/BW were not different between the two groups. Conversely, LV mass was significantly decreased (− 7%) and LV chamber volume markedly increased (+ 30%), causing a reduction in LV mass/chamber volume (− 23%; *p* = 0.07), in TiO_2_-NP rats in comparison with CTRL (Table 2). In addition, Masson’s trichrome staining demonstrated that TiO_2_-NP rats displayed an increased volume fraction of total fibrosis in the LV myocardium (Fig. 5) mostly due to a significant increase in diffuse fibrosis (+ 64%; Table 2).

**Table 2:** Tibial length, cardiac anatomy and morphometric analysis

	CTRL (*n* = 9)	TiO_2_-NP (*n* = 11)
Tibial length (mm)	38.2 ± 0.95	35.7 ± 0.25 *
BW (g)	359.3 ± 6.47	356.1 ± 5.70
LVW (mg)	1200 ± 25.50	1119.13 ± 27.91 *
LVW/BW (mg/g)	3.39 ± 0.10	3.16 ± 0.11
LV mass (mm^3^)	1132.1 ± 24.06	1055.8 ± 26.33 *
LV wall thickness (mm)	2.28 ± 0.09	2.26 ± 0.06
LV chamber diameter (mm)	7.43 ± 0.40	7.68 ± 0.25
LV chamber length (mm)	15.44 ± 0.50	15.11 ± 0.42
LV chamber volume (mm^3^)	361.3 ± 38.16	469.3 ± 31.29 *
LV mass/chamber volume	3.11 ± 0.33	2.39 ± 0.16
Total fibrosis (%)	7.64 ± 1.11	15.44 ± 3.09 *
Diffuse Fibrosis (%)	4.24 ± 0.82	11.69 ± 3.18 *
Perivascular Fibrosis (%)	3.59 ± 0.49	4.69 ± 0.45

Values are means ± SEM

Unpaired Student’s t-test

*BW* body weight measured before sacrifice, *LV* left ventricular, *LVW* left ventricular weight

*p < 0.05 vs CTRL

### Titanium dioxide quantification

We demonstrated that at the final stage (7th week), the lung level of TiO_2_ increased by about 280% as compared with the value measured after the first intra-tracheal instillation (mean values: 1st week 1093 ± 571 ppm; 7th week 4232 ± 1403 ppm, *p* < 0.05) while TiO_2_-NP_S_ were practically absent in trachea (mean values: 1st week 900 ± 230 ppm; 7th absent). Conversely, heart tissue TiO_2_ level remained high, although without a definite accumulation (mean values: 1st week 156 ± 37 ppm; 7th week 116 ± 16 ppm, n.s.)

### Titanium dioxide detection

**Fig. 6:**
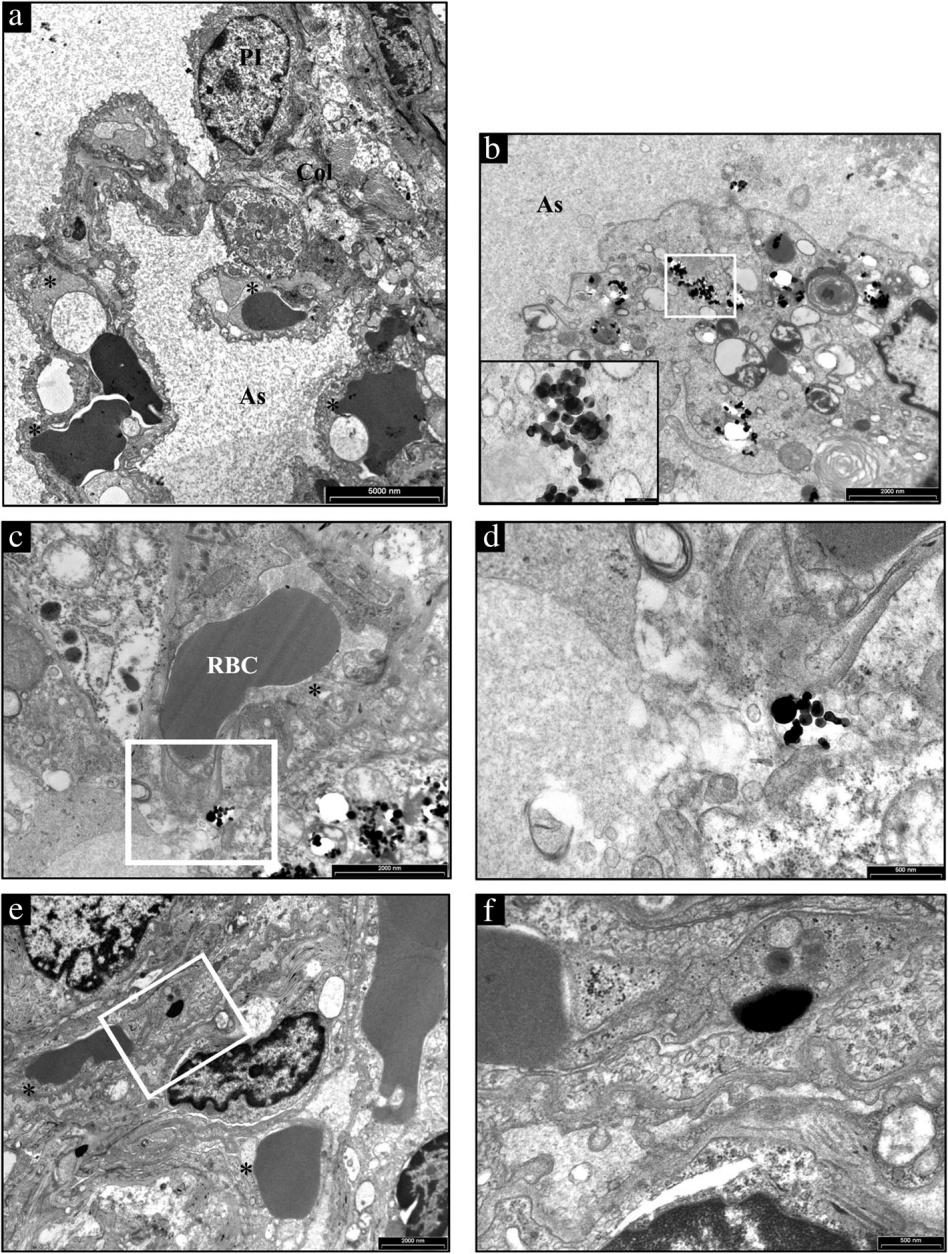
Ultrastructural features of the alveolar lung parenchyma from a representative untreated CTRL and TiO_2_-NPs treated SHR. **a** a type I pneumocyte (PI) and three capillaries (*) are present in the alveolar septum separating air spaces (As) in CTRL lung. **b** several TiO_2_-NPs are present in the cytoplasm of a large alveolar macrophage showing, in addition to vacuoles and microvescicles, onionskin-like multimembrane ultrastructures suggestive of autophagy. The area inscribed by white rectangle is shown at higher magnification in the inset. **c** an aggregate of electrondense TiO_2_-NPs is located nearby an endothelial cell lining the lumen of a capillary recognizable by the red blood cell (RBC). The white rectangle inscribes an area shown at higher magnification in **d** to document that TiO_2_-NPs are not yet internalized. **e** internalization of TiO_2_-NPs in a capillary endothelial cell that is better appreciable at higher magnification in (**f**). Scale Bars: **a** 5 μm, **b**, **c**, **e**: 2 μm, **d**, **f**: 500 nm

**Fig. 7:**
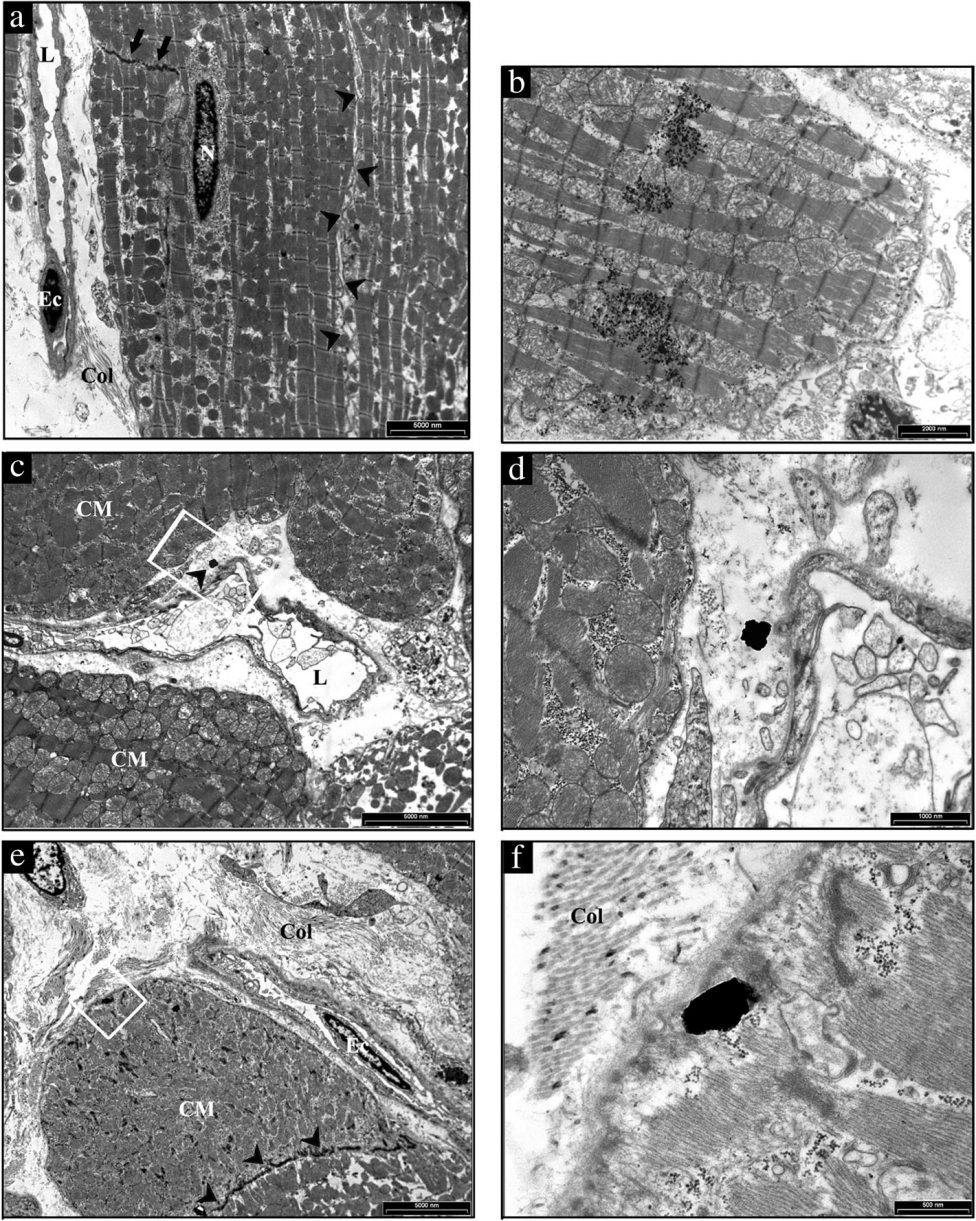
TEM analysis of the heart from a representative untreated CTRL and TiO_2_-NP treated SHR. **a** low magnification image of a CTRL heart to illustrate the sarcolemma (arrowheads) lining the surface and gap junctions (arrows) delimiting cardiomyocytes filled of mitochondria and myofibrils. On the left, collagen bundles (Col) are present in the interstitial space between an endothelial cell (Ec) lining a capillary lumen (L) and cardiomyocytes. N: cardiomyocyte nucleus. **b** aggregates of TiO_2_-NPs within the cardiomyocyte cytoplasm showing effaced myofibrils and swollen mitochondria. **c** the white rectangle inscribes an area shown at higher magnification in (**d**) in which the arrowhead points to NP located in the interstitial space between the vessel wall inscribing a lumen (L) and cardiomyocytes (CM). **e** low magnification image of a treated SHR myocardium to illustrate widening of the interstitial space by abundant fibrotic deposition (Col) surrounding cardiomyocytes (CM) and an endothelial cell (Ec) lining a capillary. Arrowheads point to gap junctions delimiting two CMs. The white rectangle inscribes an area shown at higher magnification in **f** to document the internalization of TiO_2_-NP located in proximity of the CM sarcolemma bordered by collagen bundles (Col). Scale Bars: A, C, E: 5 μm, B: 2 μm, D: 1 μm, F: 500 nm

We found the presence of TiO_2_-NPs in both, the lungs (Fig. 6) and the hearts (Fig. 7). In particular, several NPs aggregates were found free in the alveolar space (Additional file 2: Figure S2), in the interstitial space surrounding alveolar capillary (Fig. 6c and d) and inside the cells (Fig. 6e and f) as in alveolar cells displaying macrophagic/autophagic features (Fig. 6b). Morphologic evidence provided by Transmission Electron Microscopy (TEM) suggested that in the heart NPs left the capillary lumen localizing in the interstitial perivascular space (Fig. 7b-d), penetrated the sarcolemma and reached the myoplasm by establishing intimate contact with myofibrils and mitochondria (Fig. 7e and f).

### Oxidative stress and inflammation

**Fig. 8:**
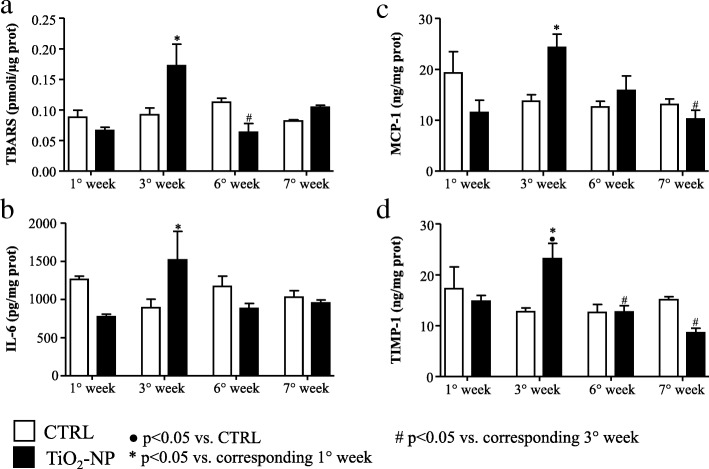
Inflammation and toxicological markers in the heart tissue. Lipid peroxidation products and different inflammatory and toxicological markers evaluated in CTRL (white bars) and TiO_2_-NPs treated (black bars) animals. **a** TBARS evaluation in the heart tissue. **b** IL-6 evaluation in the heart tissue r. **c** MCP-1 evaluation in the heart tissue. **d** TIMP-1 evaluation in the heart tissue. Two-way ANOVA (post hoc analyses: Bonferroni test) was performed and statistical significance was set at *p* < 0.05. ● vs CTRL; * vs corresponding 1° week; # vs corresponding 3° week. Data are represented as mean ± SEM

Lipid peroxidation analysis in cardiac and lung tissues demonstrated that at the 3rd week the Thiobarbituric acid reactive substances (TBARS) level significantly increased in TiO_2_-NP rats compared to the 1st week of treatment, displaying the maximum value (Fig. 8a, Additional file 3: Figure S3). Similarly, significant alterations were observed in cardiac tissue at the 3rd week for Interleukin-6 (IL-6) and Monocyte Chemoattractant Protein-1 (MCP-1) (Fig. 8b and c). To note, tissue inhibitor metalloproteinase 1 (TIMP-1) levels increased as well at the 3rd week (Fig. 8d). The treatment induced approx. 50% increment of all cytokines at the 3rd week although the difference became statistically significant only for TIMP-1.

### Gene expression analysis

**Fig. 9:**
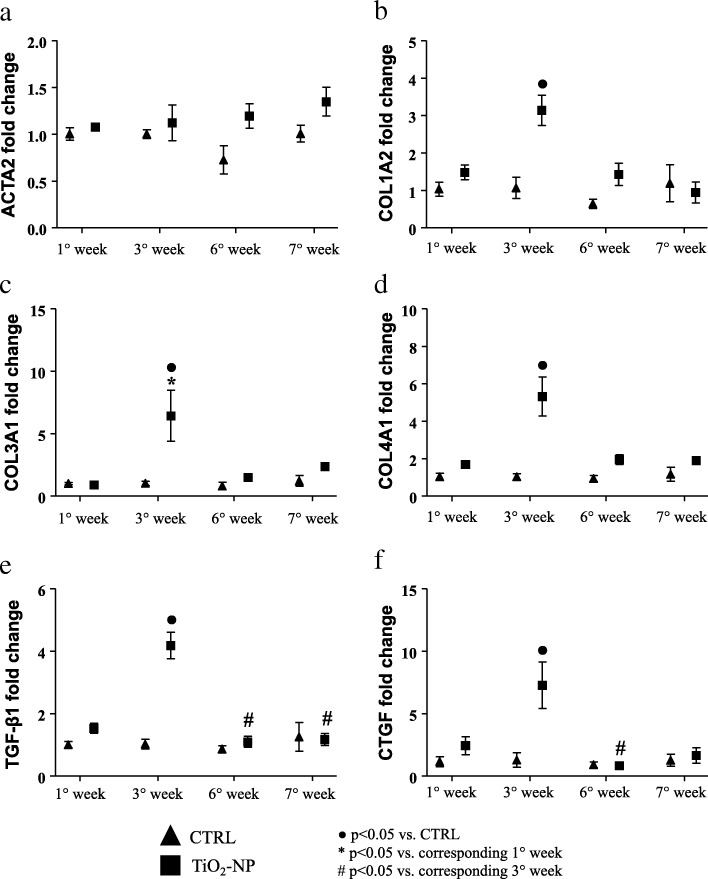
Molecular analysis displaying different gene expression linked to fibrosis deposition. Graph of different gene expression in CRTL (triangles) and TiO_2_-NPs treated (square) animals. **a** ACTA2 gene expression; **b** COL1A2 gene expression; **c** COL3A1 gene expression; **d** COL4A1 gene expression; **e** TGF-β1 gene expression; **f** CTGF gene expression. Kruskal-Wallis (post hoc analyses: Dunn’s multiple comparison) was performed and statistical significance was set at *p* < 0.05. ● vs CTRL; * vs corresponding 1° week; # vs corresponding 3° week. Data are represented as median and IQR

The expression of different genes related to myocardial fibrosis was assessed in the rat hearts, revealing that at the 3rd week TiO_2_-NPs instillation the expression of different collagen isoform genes (namely COL1A2, COL3A1, COL4A1) were definitively upregulated in treated animals compared to CTRL (Fig. 9b-d). Interestingly, at the same time point, also TGF-β1 (transforming growth factor beta-1) and CTGF (connective tissue growth factor) showed significantly increased expression (Fig. 9e and f).

## Discussion

Inhaled NPs are on the current concern for their effect on the cardiovascular system after translocation from the air-blood barrier [23]. Evidences are provided for inhaled NPs accumulation in the sites of vascular lesions [24], lymph nodes, liver, kidneys [25] and the heart [4].

Ambient (indoor) air pollution, due to the rapid urbanization worldwide, is known to be an important trigger of cardiovascular diseases especially for the people/workers that exposed continuously to NPs emitting sources with poor ventilation [26]. Following our recent findings that showed inhaled TiO_2_ NPs in the heart [19], recent literature have shown that inhaled titanium dioxide NPs activate a plethora of cardiovascular effects, i.e., activation of complement cascade in the heart [27], induction of cytotoxicity in cardiomyoblast [28], microvascular and mitochondrial dysfunction in progeny of female SD rats exposed to TiO_2_ [29], induction of myocarditis [30] and depression diastolic function in response to adrenergic stimuli [31].

In this study, we observed two concomitant responses in terms of both electrical remodeling (reversible) and mechanical and structural remodeling (irreversible) in SHRs subjected to 2 mg/kg TiO_2_ weekly repeated tracheal instillation compared with saline treated animals.

### Electrical remodeling

The electrical remodeling is related to a direct interaction between TiO_2_-NPs and cardiac cell membrane. The electrical remodelling and the arrhythmic events are related to a direct interaction between TiO_2_-NPs and cardiac cell membrane via ionic leakage as previously described [19]. The almost complete recovery of electrical functional properties, can be explain by the recruitment and presence of myofibroblasts that can concur in entrapping TiO_2_-NPs in the interstitial regions and thus reduced the amount of NPs passing the cardiomyocytes cell membrane (cf. Figure 7d). Because of that, the subsequent depolarization is now minimal but enough for enhancing excitability. The latter is the base of the biophysical phenomena of supernormal (increment) conduction velocity as described by us in fibrotic engineered in-vitro cardiac tissue [32] and in normotensive animals subjected to a single instillation of TiO_2_-NPs [19].

Noteworthy, in treated SHRs, only the number of hypokinetic arrhythmic events, including SP events (correlating with altered P wave duration) and AV blocks, as well as inducible arrhythmic events significantly increased at the 6th week and completely terminated at the end of the treatment. This scenario is presumably due to cardiac tissue damage associated with a direct acute effect of NPs, further confirmed here by ultrastructural analysis. Importantly, the number of arrhythmic events in the treated animals (sinus pauses and AV blocks) was significantly reduced approaching the values observed in CTRL (saline, positive control).

Conversely, neither the NPs treatment nor the subchronic exposure influenced QRS complex. Nevertheless, RR variability increased in both groups at the end of treatment, suggesting a time-dependency effect of this parameters independently of the treatment.

### Mechanical and structural irreversible remodeling

The irreversible mechanical and structural remodeling is related to the subchronic exposure to TiO_2_-NPs that causes in SHRs a series of maladaptive cardiac responses mainly characterized by inflammation, upregulation of genes promoting collagen deposition and fibrosis, ultimately leading to alterations in hemodynamic performance.

Electrocardiographic measurements in freely moving animals demonstrated a time-dependency for P wave duration (6th and 7th weeks) and RR interval (7th weeks). We are unable to interpret this finding and further specific atrial electrophysiological and morphological measurements are required.

Epicardial multiple lead recording demonstrated a decrease in the CVl only after the first NPs instillation, possibly due to a massive depolarization of the resting potential created by the NPs entry in the cardiac tissue [20]. It is known that SHR cardiomyocytes have a reduction in IK_1_ current responsible for diastolic membrane potential depolarization [33], and thus modulating cardiac excitability. Moreover, the treated animals denoted a major time-dependency because at the 6th week, we observed a significant increment in CVl and CVt in the treated group vs. CTRL, at the first and third week. Such increment disappeared at the 7th week after the “washout” period. The increment at the 6th week can be explained if we take into account two factors: i) the CVs are calculated only for portions of the epicardial multiple lead arrays that denoted absence of conduction blocks and ii) a role exerted by the cardiac myofibroblasts that are populating the fibrotic tissue.

Repeated exposure to TiO_2_-NPs doubled the total amount of cardiac fibrosis and triplicated the diffuse fibrosis known to fasten the progression towards heart failure [34]. These findings are in agreement with the significant upregulation of genes involved in (myo)fibroblast recruitment and collagen protein expression: TGF-β1 and COL1A2 (threefold increment), COL3A1 and CTGF (sixfold increment), COL4A1 (fivefold increment). Those genes were upregulated exactly at the third week of instillation, when also the inflammatory response initiated in the heart. Indeed, the third week of exposure is the critical time-point, when we observed the increment in oxidative stress markers and cytokines in cardiac tissue in agreement with Gurguiera et al. [35] showing that heart oxidative stress significantly correlated with inhaled titanium compared with others elemental components. Tissue inhibitor of metalloproteinase-1 (TIMP-1), known to promote myocardial fibrosis [36, 37], was also significantly upregulated at the third week compared to its control at the same time and compared to the 1st week of treatment, consistently with the significant upregulation of the related collagen-isoform genes.

Overall, the co-existence of hypertension and nanoparticle exposure leading to structural tissue damage can be responsible for the majority of alterations in gross cardiac characteristics, including chamber dilation and reduction in LV mass, as well as worsening of cardiac hemodynamic performance.

## Conclusions

The comorbidity of hypertension and inhaled nanoparticles has been largely studied in terms of hazard at epidemiological and meta-analysis levels. We provide here, for the first time, a mechanistic evidence that this co-morbidity induces irreversible hemodynamic impairment associated with cardiac structural damage, while electrophysiological alterations are mostly reversible. Translating this animal-based data to human hypertension is questionable due to species-related differences, different adaptation to the pathology and treatment, and the procedure we adopted for tracheal administration. Nevertheless, we provided here experimental evidence that the most produced engineered nanomaterial, considered to be inert, activates functional and structural remodelling of the hypertensive heart, potentially fastening the progression towards cardiac failure. Specifically, our results indicate that prolonged TiO_2_-NP exposure may reach a point of no-return (third week of treatment), in terms of myocardial tissue damage and reduces cardiac mechanical efficiency. This needs to be considered for the vulnerable subjects/workers daily exposed to nanomaterials.

## Limitations

Invasive hemodynamic and morphological measurements have been acquired only at the end of the treatment. On the other hand, it should be considered that the upregulation of genes promoting fibrotic damage and inflammation occurred at the third week and this finding prompted us to focus on the consequent later morphofunctional irreversible changes.

In this study, we limited our investigation to the ventricular cardiac fibrosis as one of the leading causes towards heart failure, in both NP-treated and untreated SHR. However, similar investigations need to be addressed for a plethora of hypertension-related diseases including atrial dysfunction, vascular endothelial dysfunction and pulmonary responses and clearance, known to be primary barriers for the inhalator route of nanoparticles. Lastly, we included here only one risk factor (i.e. hypertension) impacting only for a minimal period of the life course (adulthood).

## Methods

### Experimental animals

Experiments were conducted on sixty-four 12-week-old Spontaneously Hypertensive male rats (Envigo, Huntingdon, United Kingdom, SHR, Harlan Laboratories s.r.l., Italy) singly housed with the light on between 7 a.m. and 7 p.m. in a temperature-controlled room at 22–24 °C. The bedding of the cages consisted of wood shavings with food and water available ad libitum. All surgery procedures and experiments, when specified, were performed under anesthesia which consisted of a mixture of ketamine chloride (40 mg/kg ip; Imalgene, Merial, Milano, Italy) and medetomidine hydrochloride (0.15 mg/kg ip; Domitor, Pfizer Italia s.r.l., Latina, Italy). The period of study was selected by taking into consideration the frailty of SHR animals and their suffering due to the hypertensive condition. This study was carried out in accordance with the recommendations in the Guide for the Care and Use of Laboratory Animals of the National Institute of Health (Bethesda, MD, USA, revised 1996). The protocol was approved by the Veterinary Animal Care and Use Committee of the University of Parma (Permit: n. PMS 53/2009) and conforms to the National Ethical Guidelines of the Italian Ministry of Health. All effort was made to minimize suffering. We noticed 15% of spontaneous animal loss during the repeated anesthesia procedures from the 3rd week onwards because of the SHR hyperreactivity [38]. We did not observe animal loss for the surgical procedure.

### Particle suspension

TiO_2_-NPs (Titanium (IV) oxide, code 677469, Sigma-Aldrich, Milan, Italy) were previously characterized by us [19, 39]. Nanoparticles were suspended in physiological saline solution (10 mg/mL, stock solution). Immediately before the experiments, the suspension was vortexed and immersed in a sonication bath (Branson Ultrasonics, Danbury, CT, USA) for 5 min at 37 °C in order to minimize particle aggregation.

### Intra-tracheal instillation

Although inhalation studies are considered to be the gold standard, data from intra-tracheal instillation studies can be used for hazard assessment [16]. The instillation process was extensively described in our previous work [19]. Briefly, after anesthesia, a 16-gauge catheter was gently inserted into the trachea of rats in order to deliver 20 μL/100 g BW of saline solution or saline solution plus 2 mg/kg TiO_2_ by means of a laboratory bench P200 pipette (Gilson, Dunstable, UK). We adopted repeated weekly doses of 2 mg/kg by taking into consideration the NIOSH recommendation (https://www.cdc.gov/niosh/docs/2011-160/pdfs/2011-160.pdf) of 0.3 mg/m^3^ of TiO_2_ NPs exposure as time-weighted average concentration for 10 h per day during 40-h work week and the species-specificity [19]. With such a concentration we are further beneath the maximal exposure limit for both human [40] and rats [41–43]. Furthermore, an empty sterile syringe (1 mL) was used to gradually inflate the lung with air twice prior to the connection of the cannula port to a ventilator (Rodent ventilator UB 7025, Ugo Basile, Comerio, Italy) set to 85 cycle/min. Finally, administration of 0.15 mg/kg atipamezole hydrochloride (Antisedan, Pfizer, Milan, Italy) has been performed to wake up the animal. This protocol was repeated consecutively once a week for 6 weeks. A six-week period was selected because the SHR at this stage of age had an average systolic blood pressure of 200 mmHg, and we choose 7 weeks as longitudinal study in order to avoid physical suffering due to high systolic blood pressure and aged related cardiac implication [44].

### Outline of the experimental protocols

#### Telemetry-ECG, hemodynamic, cardiac anatomy and morphometry studies

The experimental design and measurements performed in the two experimental groups are summarized in Fig. 1. Sixteen rats were chronically instrumented with a telemetry-ECG system to enable the evaluation of arrhythmia vulnerability and the duration of the different ECG waves and intervals, in conscious freely moving animals. One week later, the animals started the experimental instillation protocol which consisted in a weekly intra-tracheal instillation of saline solution (CTRL group, *n* = 8) or saline solution added with TiO_2_ at a final concentration of 2 mg/kg (TiO_2_-NP group, *n* = 8), as described above. Four hours later, a 30-min telemetry-ECG recording was performed while the experimental animal was left alone and undisturbed in its cage. ECG recordings were also performed at the 3rd week, 6th week, and at the end of the experimental protocol (7th week) 1 week after the last intra-tracheal instillation (Fig. 1). Afterward, hemodynamic data were invasively collected in all animals and in additional 3 TiO_2_-NP and 1 CTRL rats for a total of 11 TiO_2_-NP and 9 CTRL. Finally, at sacrifice, the hearts were perfusion fixed for anatomical and structural studies.

#### Epicardial multiple-lead recording, PIXE, and molecular studies

In 38 rats belonging to CTRL (*n* = 16) and TiO_2_-NP (*n* = 22) groups, electrophysiological investigation was performed by epicardial multiple-lead recording at the same time points specified above for telemetry-ECG recordings. At death, for each time point, fresh preparations of the excised hearts and lungs were used for molecular analyses (real time PCR: *n* = 3 CTRL, *n* = 5 TiO_2_-NP), immunological assay (*n* = 3 CTRL and *n* = 4 TiO_2_-NP) while for TiO_2_-NP quantification (PIXE, see specific section below for details) the analysis has been performed at the 1st week (*n* = 3 TiO_2_-NP) and at the end of the treatment, 7th week (*n* = 2 TiO_2_-NP).

#### Transmission electron microscopy study

In 3 CTRL and 3 TiO_2_-NP rats, heart and lung samples were analyzed by TEM in order to document the presence of NPs within these tissues at the end of the experimental protocol (7th week), 1 week after the 6th intra-tracheal instillation, given that we have already documented the presence of TiO_2_-NPs in both tissues 4 hours after tracheal instillation [15].

### Animal instrumentation for telemetry-ECG recording

After anesthesia, animals were chronically instrumented with a miniaturized transmitter for telemetry-ECG recording (model TA11CTA-F40, Data Sciences International, St. Paul, MN, USA). The details of the surgical procedure have been already published by us [45]. Briefly, a ventral celiotomy was performed and the body of the transmitter (25x15x8mm) was placed in the abdominal cavity. One recording lead was fixed to the dorsal aspect of the xiphoid process, close to the apex of the heart. The other lead was subcutaneously tunneled on the thorax toward the upper insertion of the sternohyoid muscle. Here, the wire was formed into a U shape, pushed under the muscle and then along the trachea into the anterior mediastinum, to get the tip of the recording lead close to the right atrium. Finally, the muscle and skin layers were separately sutured. After surgery, all animals were given Antisedan 0.15 mg/kg im (Pfizer), flunixin 5 mg/kg im (Finadyne, Schering-Plough s.p.a, Milan, Italy), gentamicin sulphate 10 mg/kg im (Aagent, Fatro, Milan, Italy), and kept warm with infrared lamp radiation. Antibiotic therapy was continued in the following 3 days while the animals were individually housed. Rats were allowed to recover for 7 days before the onset of the instillation protocol and ECG acquisition.

### Telemetry-ECG data acquisition and processing

ECG signals were collected by a receiver (model CTR85-SA, Data Sciences International, St. Paul, MN, USA) placed under the housing cage, monitored on an oscilloscope and simultaneously routed to a personal computer, via an analog-to-digital conversion board (12 bits, 1 kHz sampling rate), for a permanent storage. The data were analyzed by using a software package developed in our laboratory, in order to measure:

i. RR interval duration, the time interval between two consecutive R wave peaks;
ii. P wave duration (atrial depolarization), between the onset and offset of P wave;
iii. PQ interval, the time interval between the onset of P wave and the onset of QRS complex (ventricular depolarization);
iv. QRS complex duration;
v. QT interval, the time interval between the onset of QRS complex and the end of T wave (ventricular repolarization);
vi. QTc interval, heart rate-corrected QT interval (Bazett’s formula);
vii. Heart rate variability. The indexes of heart rate variability included: the SDRR and r-MSSD, which quantify the state of the balance between sympathetic and parasympathetic activities on the heart and the influence on heart rate of the parasympathetic branch respectively [46];
viii. Number and type of arrhythmic events. In detail, we recorded both, supraventricular (Supraventricular Extrasystole and Sinus Arrhythmia, SVA; SP) and ventricular (AV block; Ventricular Extrasystole, VE) events.

For the evaluation of ECG wave and interval duration, 30 measures for each animal were performed.

### Hemodynamic study

After anesthesia, the right carotid was cannulated with a microtip pressure transducer catheter (Millar SPC-320; Millar Instruments, Houston, TX, U.S.A.) connected to a recording system (Power Laboratory ML 845/4 channels; 2Biological Instruments, Besozzo, Italy), and systolic and diastolic arterial blood pressures were determined. The catheter was then advanced into the left ventricle to measure (software package CHART B4.2): LVSP; LVEDP; +dP/dt_max_ and -dP/dt_max_, taken as indexes of ventricular mechanical efficiency; IVCT.

### Tibial length, cardiac anatomy and morphometric analysis

At the end of the hemodynamic procedure, the heart was arrested in diastole by injecting 5 mL of cadmium chloride solution (100 mmol/L, iv), and excised. The right ventricle and the left ventricle inclusive of the septum were separately weighed and fixed in 10% buffered formalin solution. In all animals, the length of the tibia was also determined. The volume of the LV myocardium was computed by dividing LV weight by the specific gravity of the tissue (1.06 g/mL). LV chamber length was measured from the apex to the aortic valve. The equatorial transverse section of the left ventricle (1 mm thick), cut perpendicularly to the major axis, allowed the morphometric computation of the LV equatorial diameter and wall thickness (Image Pro-plus, Media Cybernetics, Bethesda, MD, USA, version 7.0). The LV chamber volume was then calculated according to the Dodge equation, which equalizes the ventricular cavity to an ellipsoid [47]. The section was finally embedded in paraffin. Five-micrometer-thick serial sections were obtained from the equatorial slice and used for morphometric analyses. One section, stained with blue aniline Masson’s trichrome, was analyzed by optical microscopy (magnification X 250) to evaluate the volume fraction of perivascular and interstitial fibrosis in the LV myocardium. According to a procedure previously described [48], for each section a quantitative evaluation of the fibrotic tissue was performed in 60 consecutive fields from subendocardium, midmyocardium, and subepicardium with the aid of a grid defining a tissue area of 0.160mm^2^ and containing 42 sampling points, each covering an area of 0.0038mm^2^. To define the volume fraction of fibrosis, the number of points overlying myocardial fibrosis was counted and expressed as percentage of the total number of points explored.

### Epicardial multiple-lead recording

In rats under anesthesia and artificial respiration, the heart was exposed through a longitudinal sternotomy and suspended in a pericardial cradle. Body temperature was maintained with infrared lamp radiation. An 8 × 8 row electrode matrix with 1-mm inter-nodal resolution was fabricated from surgical cotton gauze. The electrode array was positioned in order to cover part of the anterior surface of the right and left ventricles [49], and the following measurements were carried out: refractoriness (by means of S1-S2 protocol) and inducible ectopic activity (*n* = 8 for each animal), ventricular excitability (Rheobase and Chronaxie, *n* = 5 for each animal and Threshold intensity for a 1 ms duration impulse, *n* = 13) and conduction velocities (CVl and CVt, *n* = 10 for each animal, as well as their ratio), as previously described [19]. Moreover, arrhythmia inducibility was evaluated as percentage of electrodes showing at least one ventricular extrasystole during effective refractory period (ERP) protocol.

### Titanium dioxide quantification by PIXE

The trachea, lungs, and heart were prepared into pellets following the previously described procedure [50]. Briefly, biological samples were dried in a 37 °C oven for 24 h, then they were freeze dried and chromium nitride (Cr_2_N) powder (Goodfellow SARL, Lille, France) was added at 8.5 ± 1.5 wt.%. Biological samples and Cr_2_N powder were ball milled to produce a homogeneous powder mixture which was hard pressed into a 2 cm diameter, 1-2 mm thick pellet. We added Cr_2_N in order to avoid charge accumulation in the sample during ion beam irradiation and have an internal beam monitor for quantitative measurements. In addition, Cr_2_N does not interfere with the measurement of other elements, because it is neither present in the biological matter nor as an impurity in TiO_2_.

PIXE measurements were performed with the University of Namur ALTAÏS accelerator (Accélérateur Linéaire Tandetron pour l’Analyse et l’Implantation des Solides). PIXE is an ion beam technique, whose physical principles can be found extensively explained elsewhere [51], providing precise quantification of chemical elements in toxicological assessments [52]. PIXE and Rutherford Back-scattering (RBS) measurements were done simultaneously with an incident ion beam of 2.5 MeV protons with an intensity of 1–1.5 nA. With respect to the beam direction, the sample was tilted at 45°, a Canberra LEGe (Low Energy Germanium) detector was located at 90° for PIXE measurements (variable solid angle up to 35 msr), and a Canberra PIPS (Passivated Implanted Planar Silicon) detector was positioned at 145° for RBS measurements (solid angle of 23.8 msr). An aluminum collimator (1 mm aperture, 0.2 mm thick) was used in front of the PIXE detector to equalize the ratio of low-energy and high-energy X-rays. The samples were mounted on a rotating device providing a total analyzed area of 140.5 mm^2^. RBS was used to derive the number of incident ions, and to quantify carbon, oxygen and nitrogen contents for matrix (lung tissue) correction of X-ray absorption effects. A BCR-126 lead glass standard was used to calibrate both detectors. The PIXE setup allowed for a detection limit of Ti in biological matrices of 10 wt.ppm [52].

Data were analyzed with the GUPIXWIN software [53]. The concentration of TiO_2_-NPs in tissues was calculated from the Ti X-ray emission, then converted to TiO_2_ assuming a 1:2 ratio of Ti:O.

### Oxidative stress and inflammation

Tissue samples were washed with PBS and included in cryovials prior to freezing at − 80 °C. Frozen tissue samples (lungs, and heart) were homogenized and sonicated in PBS supplemented with protease inhibitor cocktail (Sigma-Aldrich, Milan, Italy). Insoluble debris was pelleted and supernatants were analyzed.

Lipid peroxidation was evaluated by TBARS method, using Malondialdehyde as a standard for the calibration curve [39]. The fluorescence intensity was measured by Cary Eclipse fluorescence spectrophotometer (Varian/Agilent, Santa Clara, CA, USA) (excitation 515 nm, emission 545 nm). Four independent experiments were carried out and TBARS values were normalized to protein concentrations. Commercially available enzyme-linked immunosorbent assays (ELISA, Cloud-Clone Corp., Katy, TX, USA) were used to evaluate biomarkers of tissue inflammation (IL-6 and MCP-1), and tissue remodeling (TIMP-1). All data were related to protein concentration.

### Real-time PCR analysis

Frozen heart samples were kept in dry ice and cut into small pieces of about 50–70 mg using a scalpel. TRIzol reagent (1 ml) (Invitrogen, Thermos Fisher Scientific) was then added to each sample, and the tissue was homogenized using the TeSeE PRECESS 24 (BioRad) performing three agitation cycles at 5100 rpm for 10s at 4 °C. Homogenized tissues were centrifuged at 12000×g for 1 min, in order to remove particulate debris. The RNA was subsequently extracted from the supernatants using the kit Direct-zol RNA MiniPrep (Zymo research), following manufacturer’s instructions.

RNA concentration and quality was assessed by NanoDrop 1000 spectrophotometer (Thermo fisher Scientific): only samples with a value of A260/280 > 1.8 and A260/230 > 1.8 were used for subsequent gene expression analysis. RNA integrity was evaluated with Experion electrophoresis system using the Experion™RNA StdSens Analysis Kit (Bio-Rad).

Total RNA (1 μg) was reversely-transcribed using the SuperScript VILO cDNA synthesis Kit (Thermo Fisher Scientific) according to manufacturer’s instructions.

The amplification of the cDNA was then performed using All-in-One SYBR® Green qPCR Mix (GeneCopoeia) on a CFx96 Real-Time System C1000 Thermal Cycler (BioRad). The list of primers used for cDNA amplification is reported in Additional file 4: Table S1.

Raw expression intensities of genes of interest were normalized to the Ct value of Glyceraldehyde 3-phosphate dehydrogenase (GAPDH). Relative quantitation was performed using the ∆∆Ct method in comparison with the mean expression of the same genes in CTRL animals. Fold changes in gene expression were calculated as 2 ^(ΔΔCt)^ [54].

### Titanium dioxide detection in heart and lung tissues by TEM

Heart and lung samples from 3 CTRL and 3 TiO_2_-NP groups at the 7th week of the experimental protocol, 1 week after the last intra-tracheal instillation, were analyzed by TEM in order to document the presence of NPs within these tissues. Samples were fixed in Karnovsky solution (4% formaldehyde, 5% glutaraldehyde) for 3 h at room temperature. After washing several times with 0.1 M phosphate buffer, pH 7.2, the tissues were post-fixed in 1% osmium tetroxide (OsO_4_) for 90 min at room temperature and dehydrated by increasing concentration of alcohol. Then, samples were washed with propylene oxide and embedded in epoxy resin. Sections of 0.5 μm thickness were stained with methylene blue and safranin to morphologically select the field of interest. Subsequently, ultrathin sections of 60-80 nm thickness were collected on a 300-mesh copper grid and, after staining with uranyl acetate and lead citrate, were qualitatively examined under a transmission electron microscope (EM 208S model, Philips, Amsterdam, The Netherlands).

### Statistical analysis

Data were expressed as median and interquartile range (IQR), except for the hemodynamic and morphometric parameters (Tables 1 and 2) and oxidative stress and inflammation (Fig. 8) that were reported as means ± standard error of the mean (SEM). Normal distribution of variables was checked by means of the Kolmogorov-Smirnov test. Statistics of variables included unpaired Student’s t-test, two-way ANOVA (post hoc analyses: Bonferroni test or Games-Howell test, when appropriate) and Kruskal-Wallis (post hoc analyses: Dunn’s multiple comparison). Prism 5.0 software (GraphPad Software) was used to assess the normality of the data and for statistical calculation. The details on the specific test used for each experiment are reported in the figure legends. Statistical significance was set at *p* < 0.05.

## Additional files

Additional file 1: **Figure S1.** Spontaneous arrhythmic events. Different examples of arrhythmic events. Both, supraventricular (Supraventricular Extrasystole and Sinus Arrhythmia, SVA; Sinus Pause, SP) and ventricular (Atrio-ventricular block, AV block; Ventricular Extrasystole, VE) events were recorded. (PDF 324 kb)

Additional file 2: **Figure S2.** TEM analysis of the alveolar lung parenchyma from a SHR rat seven weeks after intratracheal instillation of TiO2-NPs. Individual or microaggregates of small electrondense NPs are present in the air space (As) as scattered within the alveolar septum in which a type II pneumocyte (PII) is recognized. NPs are also apparent in endothelial cells lining a capillary (*) and the lumen of a larger venule containing red blood cells (RBC) polymorphonuclear (PMN) neutrophils, lymphocytes (Lym) and platelets (arrow). Magnification 1800X. Scale Bar: 10μm. (PDF 2011 kb)

Additional file 3: **Figure S3.** TBARS measurement in lungs evaluated in CTRL (white bars) and TiO2-NPs treated (black bars) animals. Two-way ANOVA (post hoc analyses: Bonferroni test) was performed and statistical significance was set at p<0.05. ● vs CTRL; * vs corresponding 1° week; # vs corresponding 3° week. Data are represented as mean ± SEM. (PDF 60 kb)

Additional file 4: **Table S1.** List of primer sequences 5′-3′ used for real-time PCR analysis. (PDF 118 kb)

## Abbreviations

+dP/dt_max_: Maximum rate of ventricular pressure rise
AV block: Atrio-ventricular block
BW: Body weight
Cr_2_N: Chromium nitride
CTGF: Connective tissue growth factor
CTRL: Control
CVl: Longitudinal conduction velocity
CVt: Transversal conduction velocity
-dP/dt_max_: Maximum rate of ventricular pressure reduction
ERP: Effective refractory period
GAPDH: Glyceraldehyde 3-phosphate dehydrogenase
IL-6: Interleukin-6
IVCT: Isovolumic contraction time
LEGe: Low energy germanium
LV: Left ventricular
LVEDP: Left ventricular end diastolic pressure
LVSP: Left ventricular systolic pressure
LVW: Left ventricular weight
MCP-1: Monocyte Chemoattractant Protein-1
NIOSH: National institute for occupational health
NPs: Nanoparticles
OsO_4_: Osmium tetroxide
PIPS: Passivated implant planar silicon
PIXE: Particle-Induced X-ray emission
PM: Particulate matter
RBS: Rutherford back scattering
r-MSSD: Root mean square of successive RR interval square differences
SDRR: Standard deviation of average RR interval
SHRS: Pontaneously hypertensive rat
SP: Sinus pause
SVA: Supraventricular Extrasystole and Sinus Arrhythmia
TBARS: Thiobarbituric acid reactive substances
TEM: Transmission Electron Microscopy
TGF-β1: Transforming growth factor beta-1
TIMP-1: Tissue inhibitor metalloproteinase 1
TiO_2_: Titanium dioxide
VE: Ventricular Extrasystole
WKY: Wistar Kyoto
