# Supplementary material for: Subchronic exposure to titanium dioxide nanoparticles modifies cardiac structure and performance in spontaneously hypertensive rats
Source: Part Fibre Toxicol. 2019 Jun 24;16:25. doi: 10.1186/s12989-019-0311-7 (PMC6591966; doi:10.1186/s12989-019-0311-7)
Supplement: Supplementary file 1 — Figure S1. Spontaneous arrhythmic events. Different examples of arrhythmic events. Both, supraventricular (Supraventricular Extrasystole and Sinus Arrhythmia, SVA; Sinus Pause, SP) and ventricular (Atrio-ventricular block, AV block; Ventricular Extrasystole, VE) events were recorded. (PDF 324 kb) [file 12989_2019_311_MOESM1_ESM.pdf]

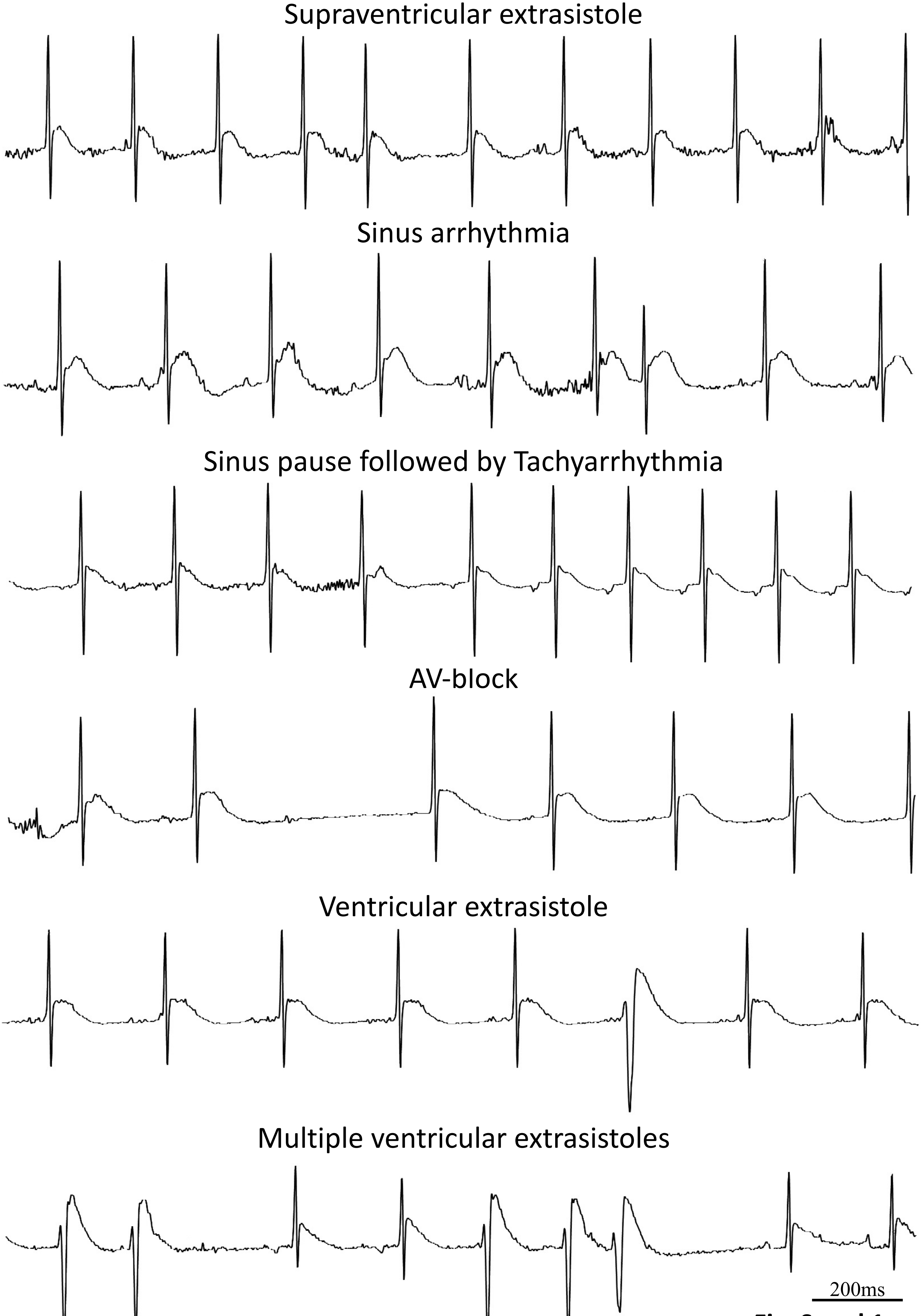
Supraventricular extrasistole
Sinus arrhythmia
Sinus pause followed by Tachyarrhythmia
AV-block
Ventricular extrasistole
Multiple ventricular extrasistoles
200ms

Fig. Suppl 1
