# Supplementary figures and images for: Subchronic exposure to titanium dioxide nanoparticles modifies cardiac structure and performance in spontaneously hypertensive rats
Source: Part Fibre Toxicol. 2019 Jun 24;16:25. doi: 10.1186/s12989-019-0311-7 (PMC6591966; doi:10.1186/s12989-019-0311-7)

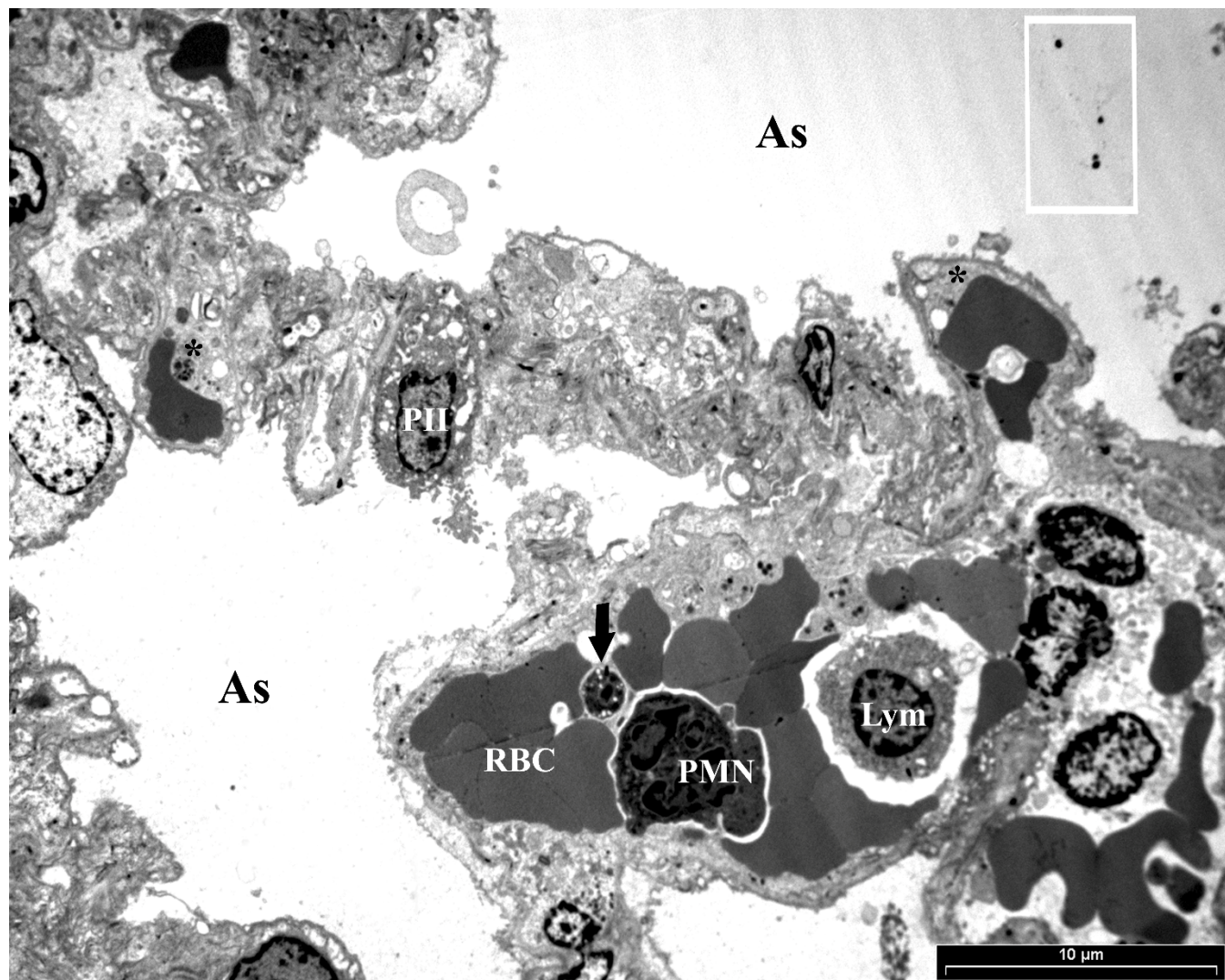

Supplement: Supplementary file 2 — Figure S2. TEM analysis of the alveolar lung parenchyma from a SHR rat seven weeks after intratracheal instillation of TiO2-NPs. Individual or microaggregates of small electrondense NPs are present in the air space (As) as scattered within the alveolar septum in which a type II pneumocyte (PII) is recognized. NPs are also apparent in endothelial cells lining a capillary (*) and the lumen of a larger venule containing red blood cells (RBC) polymorphonuclear (PMN) neutrophils, lymphocytes (Lym) and platelets (arrow). Magnification 1800X. Scale Bar: 10μm. (PDF 2011 kb) [file 12989_2019_311_MOESM2_ESM.pdf]

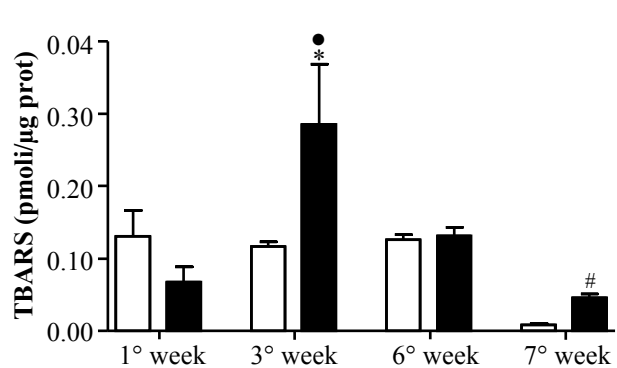

CTRL  
TiO<sub>2</sub>-NP

• p<0.05 vs. CTRL

\* p<0.05 vs. corresponding 1° week

# p<0.05 vs. corresponding 3° week

Supplement: Supplementary file 3 — Figure S3. TBARS measurement in lungs evaluated in CTRL (white bars) and TiO2-NPs treated (black bars) animals. Two-way ANOVA (post hoc analyses: Bonferroni test) was performed and statistical significance was set at p<0.05. ● vs CTRL; * vs corresponding 1° week; # vs corresponding 3° week. Data are represented as mean ± SEM. (PDF 60 kb) [file 12989_2019_311_MOESM3_ESM.pdf]
