# Supplementary material for: Subchronic exposure to titanium dioxide nanoparticles modifies cardiac structure and performance in spontaneously hypertensive rats
Source: Part Fibre Toxicol. 2019 Jun 24;16:25. doi: 10.1186/s12989-019-0311-7 (PMC6591966; doi:10.1186/s12989-019-0311-7)
Supplement: Supplementary file 4 — Table S1. List of primer sequences 5′-3′ used for real-time PCR analysis. (PDF 118 kb) [file 12989_2019_311_MOESM4_ESM.pdf]

Table S1: List of primer sequences 5´-3´ used for real-time PCR analysis.

PRIMER	SEQUENCE
GAPDH fw	5’-CCC CCA ATG TAT CCG TTG TG-3’
GAPDH rev	5’-TAG CCC AGG ATG CCC TTT AGT-3’
COL1A1 fw	5’-TAAGGGTGAAGCTGGTCCCC-3´
COL1A1 rev	5’- TTCACCACTGTTGCCTTTGG-3´
COL1A2 fw	5’-CCTCAGGGTGTTCAAGGTGG-3´
COL1A2 rev	5’- GACCACGTTCTCCTCTTGG-3´
COL3A1 fw	5’- CACAGAGGCTTTGATGGACG-3´
COL3A1 rev	5’- AACCTCACCCTTAGCACCAG-3´
COL4A1 fw	5’- TCCAGGTTTCGCTGTTCCA-3´
COL4A1 rev	5’- GCTCTCTCCTTTCTGACCTTTC-3´
TGFß1 fw	5’- ACCAAGGAGACGGAATACAGG- 3´
TGFß1 rev	5’-AGGACCTTGCTGTACTGTGT-3´
CTGF fw	5’- CCTAGCTGCCTACCGACTG- 3´
CTGF rev	5’- TTTTGCCCTTCTTAATGTTTTCC-3´
ACTA2 fw	5’- TGGAAAAGATCTGGCACCAC-3´
ACTA2 rev	5’- GAGTCCAGCACAATACCAGT- 3´
